# Fiscal Expenditures on Science and Technology and Environmental Pollution: Evidence from China

**DOI:** 10.3390/ijerph17238761

**Published:** 2020-11-25

**Authors:** Wanfang Xiong, Yan Han, M. James C. Crabbe, Xiao-Guang Yue

**Affiliations:** 1 Department of Finance, School of Economics, Huazhong University of Science and Technology, Wuhan 430074, China; xiongwf0923@hust.edu.cn; 2 School of Humanities and Social Science, Beijing Institute of Technology, Beijing 100081, China; 3 Wolfson College, Oxford University, Oxford OX2 6UD, UK; James.Crabbe@beds.ac.uk; 4 School of Life Sciences, Shanxi University, Taiyuan 030006, China; 5 Institute of Biomedical and Environmental Science & Technology, School of Biological Sciences, University of Bedfordshire, Park Square, Luton LU1 3JU, UK; 6 Department of Computer Science and Engineering, School of Sciences, European University Cyprus, Nicosia 1516, Cyprus; 7 CIICESI, ESTG, Politécnico do Porto, 4610-156 Felgueiras, Portugal; 8 Department of Business Sciences, University Giustino Fortunato, 82100 Benevento, Italy

**Keywords:** FESTs, environmental pollution, fixed asset investments, R&D

## Abstract

Studying the driving factors of environmental pollution is of great importance for China. Previous literature mainly focused on the cause of national aggregate emission changes. However, research about the effect of fiscal expenditures on science and technology (FESTs) on environmental pollution is rare. Considering the large gap among cities in China, it is necessary to investigate whether and how FESTs affect environmental pollution among cities. We adopted three kinds of typical environmental pollutants including sulfur dioxide (SO_2_) emissions, wastewater emission, and atmospheric particulate matter less than 2.5 micrometers in diameter (PM_2.5_). Using the data of 260 prefecture-level cities over ten years in China, we found that FESTs play a significantly positive role in reducing sulfur dioxide (SO_2_) emissions and PM_2.5_ concentrations, but fail to alleviate wastewater emissions. Specifically, for every 1% increase in FESTs, SO_2_ emissions were reduced by 5.317% and PM_2.5_ concentrations were reduced by 5.329%. Furthermore, we found that FESTs reduced environmental pollution by impeding fixed asset investments and by promoting research and development activities (R&D). Moreover, the impacts of FESTs on environmental pollution varied across regions and sub-periods. Our results are robust to a series of additional checks, including alternative econometric specifications, generalized method of moments (GMM) analysis and overcoming potential endogeneity with an instrumental variable. Our findings confirm that government efforts can be effective on pollution control in China. Hence, all governments should pay more attention to FESTs for sustainable development and environmental quality improvements.

## 1. Introduction

Over the past 40 years, China’s urbanization and industrialization have progressed rapidly. Simultaneously, this intensive economic development has caused serious environmental pollution. Water and air pollution in particular have seriously affected people’s quality of life and the rephrasing of sustainable regional development [1]. Based on the negative impacts of pollution on the physical and mental health of residents, scientists have listed the main components of air pollution, including solid particulate matter (PM) and sulfur dioxide (SO_2_), as the main targets for monitoring and controlling air pollution. According to the World Bank’s (2007) report, Cost of Pollution in China: Economic Estimates of Physical Damages, the damage caused by air and water pollution in China was equivalent to 5.8% of its real GDP [2]. This finding indicates that environmental pollution is a typical manifestation of negative externalities in the production process: the private marginal costs of discharging environmental pollutants are lower than the social marginal costs.

Previous literature documented that government fiscal expenditure plays a key role in environmental quality. For instance, López et al. (2011) provided a theoretical model for how both the amount and composition of government spending affected environmental pollution [3]. In line with this stream of research, Hua et al. (2018) documented that the public education spending had a negative relationship with SO_2_ emission [4]. Lin et al. (2019) maintained that fiscal spending promoted green economic growth through spending on both education and R&D [5]. However, the effects of fiscal expenditures on science and technology (FESTs) on environmental pollution have received little attention. This paper fills this gap.

López et al. (2011) modeled and measured the following four mechanisms about fiscal spending patterns on the environment [3]. First, high economic growth increased environmental pressure, and this causation is known as the scale effect. Second, human capital-intensive productions tended to pollute less than physical capital-intensive productions, which is called the composition effect. Third, investment in R&D and the diffusion of knowledge may have reduced the pollution–output ratio by improving efficiency and developing cleaner technologies, and this reduction was a result of the technique effect. Fourth, the increase in income had a positive impact on environmental quality, which is called the income effect. In this study, we investigate the effect of FESTs on environmental pollutants. In other words, we identify and analyze the technique effect.

We studied the relationship between FESTs and environmental pollution in China for the following reasons. Firstly, China is facing concerns about environmental pollution control. According to the Air Pollution Prevention Action Plan issued by the State Council in 2017, the concentration of inhalable particulate matter in cities at the prefecture or higher level should be reduced by more than 10% from the levels in 2012. The concentration of fine particulate matter in the Jing-Jin-Ji region [6], Yangtze River Delta, and Pearl River Delta should be decreased by 25%, 20%, and 15%, respectively.

Secondly, the market failure of environmental pollution provides theoretical justification for governmental intervention in environmental issues [7]. In China, most of the environmental managements are delegated to local governments [8]. Because local governments play a positive role in economic development and the coordination of social orders, they are of great importance in the implementation of environmental policies and governance. More important, China’s current Laws of Environmental Protection and Laws of the Prevention and Control of Air Pollution require local governments to take responsibility for the air quality of their regions, thus leading these governments to increase investment in the prevention and control of air pollution. Consequently, it is necessary to study the relationships between FESTs and environmental pollution.

We used prefecture-level panel data to assess how FESTs affected the concentration of SO_2_ commission, wastewater, and atmospheric particulate matter of less than 2.5 micrometers in diameter (PM_2.5_) in China from 2004 to 2015. These three pollutants are criteria pollutants, ensuring that we accessed the maximum number of standardized and consistent observations. We showed that more FESTs significantly reduced SO_2_ emissions, which is consistent with the findings of López et al. (2015) and Hua et al. (2018) [4,9], but fail to significantly alleviate PM_2.5_ and polluted water emission. Moreover, we used government R&D investments and fixed asset investments as mechanism variables to test the channels. We found that FESTs could promote R&D expenditures and impede fixed asset investments, which together could lead to a mitigation of total pollution.

An endogeneity problem arises, because it is difficult to disentangle whether serious environmental pollution leads to more FESTs or vice versa. However, Hua et al. (2018) argued that reverse causation seems improbable to bias our estimates since pollution emission of a certain year is unlikely to affect fiscal spending of the same year. The National People’s Congress and the Ministry of Finance predetermine and approve the governmental fiscal budget. FESTs can hardly be changed. Thus, FESTs can remain a major endogenous factor due to the omitted variable problem. Another possible cause of estimation bias may be the imprecision in calculation of China’s macroeconomic aggregates. The aggregation of measurement error in macroeconomics, especially in developing and transitional countries, is a well-known problem in empirical literature [10].

Following recent Hua et al. (2018) and Lin et al. (2019) [4,5], we employed an instrumental variable method and the generalized method of moments (GMM) to overcome potential endogeneity. Furthermore, we found that the effects of FESTs on reducing environmental pollution varied across regions and times, mainly due to their varying emphases on environmental pollution at different stages of economic development.

We contribute to the existing literature in two of the following ways, from theoretical and empirical aspects. Firstly, we add to the growing body of literature that analyzes the relationship between government expenditure and environmental pollution. Prior studies analyze government spending composition [3] and public education spending [4]. However, little attention has been paid to the FESTs, which is a mandatory item in the fiscal budget. Our study differs from prior researches because we focus on three kinds of underlying mechanisms including R&D, fixed asset investment and environmental pollution intensity and use micro-level city data to explore the impact of FESTs on environmental pollution. To the best of our knowledge, we are the first to examine the impact of FESTs on various environmental pollutants.

Secondly, we constructed a theoretical model to study the FESTs-environmental pollution relationship. In addition, the effect of FESTs on environmental pollution is greatly heterogenous across environmental pollution in different areas. Thus, our study provides additional insight into the existing literature, and suggests that local governments should invest more on FESTs to reduce environmental pollution.

Thirdly, this study enhances our understanding of the factors on environmental costs and ecological benefits. We found that FESTs could reduce SO_2_ emissions and PM_2.5_ concentrations, but fail to significantly alleviate polluted water emission. FESTs can play a role in guiding funds because they help to attract more external private investment, thereby contributing to the optimization of technical structure. In addition, our analysis of the impact of FESTs should be of interest to governments and regulators who are concerned with environmental pollutant and economic development.

The rest of our paper is organized as follows. In Section 2, we provide a literature review and develop the hypotheses. In Section 3, we discuss the data, variables, and econometric specifications. In Section 4, we present the baseline estimation results. In Section 5, we give a series of robustness checks, and in Section 6, we conclude the paper.

## 2. Literature Review and Developed Hypotheses

A strand of literature explores the relationship between the fiscal expenditure and environmental pollution. For instance, Jiang, et al. [11] adopted a spatial econometric model to study the direct and indirect spillover effects on provincial governments on SO_2_ emissions in China. They found that there existed an inverted U-shaped curve and expenditure for environmental protection was negatively correlated with SO_2_ pollution. Wang and Li [12] investigated the effect of financial expenditure on carbon emission by using provincial-level dynamic panel from 1996 to 2010. Their results showed that the scale of financial expenditure increased per capita carbon emissions, whereas the composition of financial expenditure reduced per capital carbon emissions.

Another strand of literatures examined the impact of fiscal policy on environmental pollution. For example, Cheng*,* et al. [13] evaluated the effect of fiscal decentralization on CO_2_ emissions in China by using a dynamic panel regression model during the 1997–2015 sample period. Their results showed that the effect of fiscal decentralization on CO_2_ emissions was nonlinear, and per capita fiscal expenditure amplified the negative relationship between fiscal decentralization and CO_2_ emissions. Consistent with Cheng, Fan, Chen, Meng, Liu, Song and Yang [13], Hao*,* et al. [14] also documented the inverted-U shaped relationship between fiscal decentralization and GDP per capita. However, these researches mostly studied the fiscal policy and the total financial expenditure on environmental pollution. Studies on FESTs on environmental pollution are rare. We have filled this gap. According to our knowledge, we are the first to study the effect of FESTs on environmental pollution.

Numerous empirical studies have shown that fiscal spending is a significant determinant of environmental pollution [3,4,9,15,16,17]. For instance, Halkos and Paizanos (2013) used data from 77 countries to examine the impacts of government spending on environmental pollution [16]. They found that government spending had a negative and direct impact on SO_2_ emissions, while the direct effect on carbon dioxide pollution was negligible. López et al. (2011), emphasized the importance of government spending structure [3]. Their study suggested that spending structures focused on public services were conducive to reducing pollution, while increasing government spending had no effect on environmental quality unless the structure of expenditure was changed. López and Islam (2015) studied the effects of federal and state government expenditures on important air pollutants in the United States [9]. Their results showed that state and central governments that redistributed spending on private goods to social and public goods could reduce air pollution concentration, while changes to the composition of federal spending had no effects on air pollution concentration.

According to the literature, the direction of the impact of fiscal expenditure on environmental pollutants is uncertain. This direction is influenced by factors such as the external characteristics of pollutants [18], the scale and structure of fiscal expenditure [19], and the efficiency of expenditure [16]. Therefore, governments cannot simply rely on increasing the scale of fiscal expenditures to reduce environmental pollution [3]. Instead, the government should identify expenditure items that are conducive to mitigating environmental protection in each classified project to reduce pollutant emissions based on the specific conditions of their own environmental pollution.

Generally, FESTs include investments in the green economy and introduce advanced emission reduction technologies, which are effective ways to reduce pollution emissions [5]. As discussed earlier about the technique effect mechanism, FESTs can accelerate the adjustment of the production factor structure through green production technology and R&D investment [4,20], and create a good external environment for enterprise-level technological innovation and thus reduce environmental pollution [16]. López et al. (2011) reported that a 10% increase in the share of public expenditure may result in a 4% reduction in SO_2_ concentration and a 7% decrease in lead concentration [8]. Hua et al. (2018) used city-level data to estimate the composition effect and the technique effect of education spending and R&D spending in China and found that the former seemed to be slightly stronger than the latter [4]. Clearly, the adjustment of the public expenditure structure can be an effective supplement to a government’s environmental regulations. In addition, we construct a theory model to document the relationship between FESTs and environmental pollution in Appendix A. In this vein, we propose the following hypothesis:

**Hypothesis** **1** **(H1).** *The effect of FESTs on environmental pollution is negative.*

Previous empirical literature has shown mixed results of how fiscal expenditures affect pollution. Traditional macroeconomic theory suggests that an increase in government spending would improve the economic operating environment and promote economic growth [21]. However, with the deepening of theoretical research, the negative correlation between government scale and economic growth has also attracted widespread attention. The expansion of government expenditure increases taxes. Taxes crowd out private-sector investments and consumptions. Therefore, government expenditures negatively affect economic developments [22].

The analysis of indirect effects also requires the determination of the shape of the environmental Kuznets curve. Many scholars claim that there is an inverse U-shaped relationship between environmental pollution and per capita real income [18,23]. Specifically, when economic development reaches a certain level, environmental pollution will be curbed by more investments in environmental protection or transformations of low-end polluting industries [24]. Yu and Chen (2010) investigated China’s provincial panel data and find that the expansion of government spending significantly influenced energy intensity since the Asian financial crisis [25,26]. The positive impact of government spending has remained significant since the changes in China’s economic conditions.

In general, FESTs guide the adjustment of the production-factor structure by accumulating human capital and providing a good external environment for enterprises to control pollution through technological innovation [4,26,27]. For instance, Lin and Zhu (2019) documented that education spending and R&D spending promoted green economic growth through human-capital intensive activities and technological activities [5], which is consistent with the results of Hua et al. (2018) [4].

Increasing the proportion of investment in clean technology and introducing advanced emission reduction technologies are effective ways to reduce pollution emissions [28]. Levinson (2015) documented the existence of technique effect by revealing the improvement value of US manufacturing output and reducing pollution [20]. Sandberg, et al. (2019) maintained that spending more on R&D and innovation could promote enterprise to adopt the production technologies [29]. In addition, increased FESTs could provide good external support for the production-technology innovation of enterprises and encourage them to introduce clean production technologies and management methods to reduce the demand for polluting resources. In this vein, combined with the theory model in Appendix A1, we propose the following hypothesis:

**Hypothesis** **2** **(H2).** *FESTs improve the environment by increasing R&D.*

Most studies ignore the willingness of governments to engage with FESTs, which results in omission errors, especially for the Chinese policy system. Fiscal policy plays a key role in the accumulation and allocation of an economy’s resources [16,30]. The classic pollution haven hypothesis (PHH) suggests that the strict enforcements of environmental regulations in developed countries increase the production costs of enterprises, thus making economically underdeveloped countries safe havens for highly polluting industries [31]. However, the Porter Hypothesis argues that environmental regulation and corporate competitiveness should be complementary rather than mutually exclusive. The results about environmental regulations on environmental pollution are mixed.

In China, FESTs are major driving forces for innovation. The 3rd Plenary Session of the 18th CPC Central Committee clearly stated that it is necessary to “improve the government’s support mechanism for basic, strategic, cutting-edge scientific research and common technology research.” [32]. Thus, local governments are more willing to inject limited financial resources into FESTs, which plays a significant role in crowding out fixed assets investment. During the past decades, large-scale investments have been the main driving force for local economic growth. New enterprises can enjoy new profitable opportunities by improving production efficiencies, i.e., the innovation compensation effects brought by FESTs [33]. Thus, the enterprises will increase green production efficiency instead of traditional large-scale investments. Third, engaging with FESTs on behalf of the government allows enterprises to cooperate with the government to reduce pollution by decreasing fixed assets investment. According to previous literature, firms can access more finances and government subsidies [34].

In addition, strict environmental regulation may create the latecomer advantage. This means that new enterprises can enjoy new profitable opportunities by improving production efficiencies, i.e., the innovation compensation effects [35,36]. In a country whose economic growth relies on governments and investments, large-scale investments are still the main driving force for local economic growth. Adjusting public expenditure structure may supplement government’s environmental regulations with lower costs. In this vein, we propose the following hypothesis:

**Hypothesis** **3** **(H3).** *FESTs improve the environment by strengthening environmental regulation, which in turn strengthen the supervision of enterprises and reduce fixed assets investments.*

## 3. Econometric Specification and Data

### 3.1. Econometric Specification

Our estimation strategy includes three steps. Firstly, we use a fixed effect (FE) panel regression to test H1, which is whether FESTs can explain environmental pollutions. The choice of a fixed effect regression over a random effect regression is based on the Hausman test, not detailed here to save space. The regression model is defined as Equation (1).

(1) $$Pollution_{i,t+1}=\;\beta_{0}+\beta_{1}FESTs_{i,t}+\beta_{2}Control_{i,t}+\delta_{i}+\theta_{t}+\varepsilon_{i,t}$$

where $Pollution_{i,t+1}$ stands for environmental pollutions, measured as the log of average annual PM_2.5_, SO_2_ concentration, and wastewater (WP) emission; $FESTs_{i,t}$ is the ratio of FESTs to total government expenditure. The subscripts i and t represent city and year, respectively. The control variables are per capita GDP (*Pgdp*), per capita GDP squared (*Pgdp*^2^), economic structure (*Es*), environmental regulation (*Eq*), foreign direct investment (*Fdi*), and openness (*Trade*). The FESTs effects on environment pollution are summarized by $\beta_{1}$. H1 predicts that FESTs curb environmental pollutions, therefore $\beta_{1}$ should be significantly negative.

Secondly, we test H2 which predicts FESTs boost both green technologies and green production efficiencies by estimating the following regression

(2) $$Rdec_{i,\;t+1}\;=\;\alpha_{0}+\alpha_{1}FESTs_{i,t}+\alpha_{2}Control_{i,t}+\delta_{i}+\theta_{t}+\varepsilon_{i,t}$$

where Rde is the ratio of R&D expenditure to total GDP. The control variables are *Pgdp*, *Es*, financial development (*Fd*), urbanization (*Ud*), *Fdi*, and *Trade*. According to H2, we expect $\alpha_{1}$ to be positive.

Thirdly, H3 proposes that $FESTs_{i,t}$ could improve the environment by strengthening the supervision of enterprises and reduce investment in fixed assets. We establish the two following regressions:

(3) $$Rfi_{i,t+1}=\gamma_{0}+\gamma_{1}FESTs_{i,t}+\gamma_{2}Control_{i,t}+\delta_{c}+\theta_{t}+\varepsilon_{i,t}$$

where $Rfi_{i,t+1}$ denote the ratio of fixed asset investments to GDP, respectively. Other variables are defined as in Equations (1) and (2). According to H3, $\gamma_{1}$ should be significantly negative. Furthermore, $\delta_{i}$ represents city fixed effects to control time-invariant city-specific factors, $\theta_{t}$ are the year fixed effects that account for macro or technological shocks to the economy by treating all cities identically, and $\varepsilon_{i,t}$ is an idiosyncratic error that is assumed to be independent and identically distributed with zero mean and fixed variance.

### 3.2. Variable Measurement

To investigate the relationship between the establishment of FESTs and environmental pollution, we construct our sample based on 260 prefecture- or higher-level cities in China from 2004 to 2015 according to the data availability. Most data are obtained from the China City Yearbook and the China Stock Market and Accounting Research Database (CSMAR). CSMAR is a leading economic database in China. In addition, we compiled the city-level PM_2.5_ concentration data, which are intensively monitored. Compared to the provincial panel data, such as those used by Auffhammer and Carson, 2008; Hao et al., 2015 [37,38], our city-level panel data have greater freedom and can reflect the essential characteristics of Chinese pollution. The expected coefficient for the dependent variable is negative. Following previous studies [4,15,39], and the availability of sample data, we selected SO_2_ (unit: ton), PM_2.5_ (μg/m^3^), and industrial waste water (unit: million ton), which are of widespread concern, as environmental pollution indicators. Investigating the determinant of environmental pollution may help the Chinese government to comprehensively and deeply understand the effect of FESTs on China’s environmental quality.

#### 3.2.1. Dependent Variable

Our first measure is SO_2_ emission, which is defined as the total amount of sulfur dioxide discharged into the atmosphere during the production process and fuel combustion process of industrial enterprises in the plant area. According to Cole [40] and Halkos and Paizanos [41], SO_2_ is a traditional industrial pollutant which is of highly concern during the industrialization of China. The SO_2_ data is from the China City Statistical Yearbook (CCSY). SO_2_ emissions is production-generated.

Following He [42], we adopt wastewater as our second measures, which is calculated as the natural logarithm of industrial wastewater emissions. Wastewater is one of the main groups of environmental pollutants. We also obtain the wastewater from the CCSY.

Our third measure is PM_2.5_ concentration. The PM_2.5_ concentration data cover a long-time span of almost all cities in China, which is crucial in accurately identifying environmental pollution. However, PM_2.5_ concentration is a mix between consumption-generated and production pollution. Specifically, we obtained the PM_2.5_ concentration data from 2004 to 2013 [43] and obtained the PM_2.5_ concentration data from 2014 to 2015 from “China Air Quality Online Testing and Analysis.” Ma et al. (2016) used the longitude and latitude raster data. These data are generated by simultaneously incorporating satellite- and ground-monitoring data into a two-stage spatial statistical model [44]. We further tune in these data byusing raster data, and optimizing the two-stage spatial statistical model. Previous literature often cited the satellite monitoring PM_2.5_ concentration data published by Columbia University’s Social and Economic Data and Application Center. The advantage of our data is that we use both indirect satellite-monitoring data and direct ground-monitoring data [33,44].

#### 3.2.2. Independent Variable

The main explanatory variable of interest is $FESTs_{i,t}$, the ratio of FESTs to total government expenditure. The FESTs data is from the CCSY. According to the Budget Law of China, expenditure on science and technology is a mandatory item in the fiscal budget. FESTs provide financial support for a variety of actions, such as the research and developments projects that are directly funded by governments and the transfer of technologies and patents, etc. Among these expenditures, the most effective way for FESTs to promote research and developments is government funding for the government funded research projects. These projects typically focus on fundamental scientific research and are more likely to have long-lasting and wide-spreading effects on the whole society. Most of these projects are mainly conducted by Chinese universities and other public funded research agencies. Like most other countries, the fiscal budgets spending schemes in China are prepared by the Ministry of Finance at the central government level and Finance Departments or Bureaus at the local government levels. Once the budgets are approved by the National or Local Peoples’ Congress (i.e., China’s legislative body), the governments need to strictly follow the budgets.

#### 3.2.3. Control Variable

Additional controls are per capita GDP (in 2004 CPI-adjusted terms; *Pgdp*); per capita GDP^2^ (*Pgdp*^2^), which is motivated by the classic environmental Kuznets hypothesis of an inverse-U relationship [24]; ratio of gross product in secondary industries to total GDP (*Es*) [45,46,47]; green-coverage rate of built-up areas (*Eq*) [48]; ratio of actual foreign investment to total GDP (*Fdi*); ratio of the sum of export and import to total GDP (*Trade*) [4,48]; ratio of loan balance in financial institutions to total GDP (*Fd*); and ratio of non-agricultural population to total population (*Ud*). The R&D intensity data are from the China Science and Technology Statistical Yearbook. All the variables are defined in Table 1.

### 3.3. Summary Statistics

Table 2 provides summary statistics of the city-level variables. The mean value of *PM_2.5_* in our sample is around 4.338, the mean value of *SO_2_* is 3.726, and the mean value of *WP* is 8.509. For pollutant intensity variables, the mean value of *DPM_2.5_* is −2.409, the mean value of *DSO_2_* is 3.832, and the mean value of *DWP* is 1.707. For control variables, the average value of fixed asset investments is 0.626 and the average value of *R&D* is about 0.029. The average value of *FESTs* is 0.012, the average value of *Pgdp* is 10.119, the average value of *Es* is 49.616, the average value of *Eq* is 0.373, the average value of *Fdi* is 0.021, the average value of *Trade* is 0.208, the average value of *Fd* is 0.772, and the average value of *Ud* is 0.737.

**Figure 1:**
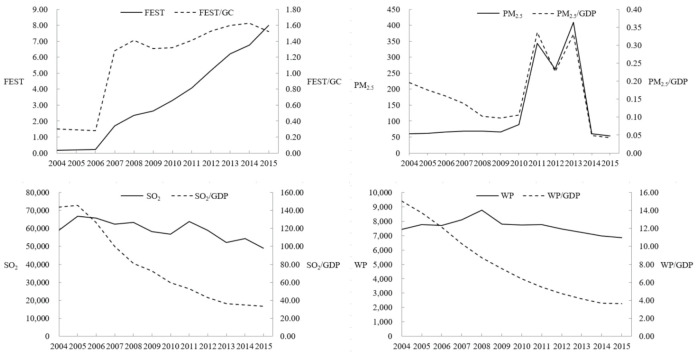
Fiscal expenditures on science and technology (FESTs) and environmental pollutions in China over 2004–2015.

Figure 1 shows the trends of FESTs, the ratio of FESTs to total government expenditures (GC), environmental pollution (PM_2.5_, WP, and SO_2_) emission intensities, and their proportions of GDP in our sample period. Through simple trend analysis, we determined characteristics of the growth trends of China’s FESTs. The growth of FESTs from 2004 to 2006 is flat, but the trend has risen at a rapid rate since 2006. Since then, there have been only minor fluctuations, and the main reason for this stability is that during the 2008 global financial crisis, countries increased government spending to stimulate economic activity. The Chinese government has also launched a four trillion investment plan to guard against the crisis. Existing research proves that fiscal expenditure is of great importance to macroeconomic growth, and economic growth is one of the most significant factors causing environmental pollution [24]. The intensity of SO_2_ and WP emission before 2006 is significantly higher than that of unit SO_2_ and WP, but the rate of emission has reversed since 2007.

We also observed a rather similar upward and downward trend in the share of PM_2.5_ and PM_2.5_/GDP. These contaminants indicate distinct characteristics and trends over time. Therefore, research on contaminants provides a more comprehensive understanding of China’s environmental quality. For example, SO_2_ is a typical industrial air pollutant that has long been under government control due to its environmental and ecological hazards [33].

## 4. Estimation Results

### 4.1. Baseline Estimation

Table 3 presents the regression results for testing H1. Columns 1–9 used PM_2.5_, SO_2_ emissions, and WP as the environmental pollution quality measures, respectively. FESTs were negatively related to the pollution measures, statistically significant at the 5% level for PM_2.5_ concentrations and statistically significant at the 1% level SO_2_ emissions. However, the coefficients to WP were insignificant. These results are also economically significant, for every 1% increase in FESTs, SO_2_ emissions were reduced by 5.317% and PM_2.5_ concentrations were reduced by 5.329%, but the FESTs could not alleviate wastewater emission. Overall, our results are consistent with H1.

Generally, cities with heavy air pollutions tend to cluster together geographically. Therefore, it is necessary to strengthen environmental regulation cooperation across cities. In terms of the nature of public goods, expenditure policy may have positive external effects [49]. A local government raising environmental protection expenditures and reducing environmental pollutant emissions will benefit surrounding cities, thus likely decreasing their willingness to invest on environmental protections. Lipscomb and Mobarak (2017) use Brazilian panel data and showed that the externalities of reducing water pollutions led to free riding behavior [50]. Sigman (2014) also examined this phenomenon due to competitions among local governments [51,52]. The impact of externalities led local governments to choose direct capital expenditures to promote economic growth and provide less public service for non-productive expenditure.

The coefficients to the controls on pollutants are not all robust. The results show that the coefficient to *Pgdp* is positive and statistically significant at the 5% level for both PM_2.5_ concentrations and SO_2_ emissions, and at the 10% level for SO_2_, while its squared effect (*Pgdp*^2^) is for PM_2.5_ concentrations and SO_2_ emissions is significantly negative at the 5% level. The inverse U-shaped curve shows that environmental pollution is intensified in the early stage of economic development but improves after reaching a certain level. Therefore, the EKC hypothesis is consistent with earlier studies of long-run development [52,53,54]. China’s central government had been evaluating local government officials mainly based on the metrics of local GDP growth rates. However, the fixation on GDP has changed recently as the central government has started to rely on diversified metrics to evaluate local government officials. The change of evaluation metrics weakens local government officials’ GDP Tournament. Therefore, pollution emissions caused by GDP growth competition have declined.

The economic structure is positively related to SO_2_ emissions, statistically significant at the 1% level. That is, industrialization enhances SO_2_ emissions. This result makes intuitive sense in that different industrial structures correspond to different pollution discharge structures. The coefficient to the *Eq* is significantly positive at the 5% level for WP, which indicates that environmental regulations reduce water qualities. The coefficient to *Fdi* is insignificant for PM_2.5_ level and SO_2_ emissions, but negatively related to WP, statistically significant at the 10% level, indicating that openness to trade is conducive to reducing air pollution. This result also shows that the pollution haven hypothesis is applicable to China [31]. The influence of foreign direct investment (FDI) on the environment depends on the combined effects of scale, structure and technology [52]. FDI is conducive to reducing pollution emissions from Chinese factories. The main reason may be that FDI can introduce and diffuse advanced technologies, making positive technical effects exceed negative scale and structural effects [55]. The coefficient to trade is insignificant.

### 4.2. Mechanism Tests

Furthermore, we ran a regression to explore the impact of FESTs on fixed asset investments and R&D expenditure (see Table 4). Consistent with H2, FESTs enhanced R&D expenditure, significant at the 10% level. This result is also consistent with the technology cleaning effect proposed by Hua et al. (2018) [4]. FESTs are major driving forces for innovation in China. The 3rd Plenary Session of the 18th CPC Central Committee clearly stated that it is necessary to “improve the government’s support mechanism for basic, strategic, cutting-edge scientific research and common technology research.” The government’s most direct means of innovation support should be financial and scientific investment in national innovation [32]. Therefore, in this paper we provide evidence for the clean technology effect of R&D investment on the environment from a fiscal perspective and show that such investment could reduce pollution through technological innovation. Our results corroborate the conclusion that the technological advancements positively affect environmental quality [24].

FESTs play a guiding role for R&D investment decisions in that they help clarify the optimization of technical structure. In general, the Chinese government has reduced the level of pollution by transforming the structure of public expenditure and by promoting spontaneous technological innovation and developing public services to promote the factor-input optimization for social production. However, critics point out two possible negative effects of FESTs. Firstly, FESTs may squeeze out individual R&D investment. Secondly, enterprises (or regions) can easily engage in low-quality tactical innovations to be eligible for the government’s financial supports of innovations. Nevertheless, most studies show that fiscal technology investment contributes to innovations [26,27].

Consistent with H3, the coefficient of FESTs for fixed-asset investment was negative and significant at the 1% level, indicating that FESTs have a restraining effect on fixed-asset investment. The negative effect of FESTs on environmental pollution reflects the government’s effort to strengthen environmental regulations, which will lead to increased production costs of polluting products, thereby reducing investment in fixed assets. The Porter Hypothesis argues that stricter environmental regulations promote corporate innovations so much so that the benefits brought about by innovations can offset or even exceed an enterprise’s compliance costs [36]. In addition, stricter environmental regulations require enterprises to improve resource utilization and management efficiency. Enterprises are also obligated to implement more stringent production standards, thereby increasing production compliance costs and reducing fixed asset investments.

## 5. Robustness Checks

### 5.1. Alternative Regression Specifications

Table 5 reports the regression results of FESTs on environmental pollution intensity. FESTs were negatively related to the pollution intensity, statistically significant at the 5% level for PM_2.5_ intensity and 1% level for SO_2_ emission intensity, respectively. Our results were consistent with the results in baseline regression.

### 5.2. Addressing Potential Endogeneity Issues

As mentioned earlier, there may be omitted variable concern from environmental pollution to FESTs. Using instruments on FESTs can alleviate possible endogeneity concern. Table 6 present the two-stage least squares (2SLS) estimates of the causal effect of FESTs on environments, by using the province average of *FESTs*. Concerning identifications, the results in the first stage suggest that the instruments are valid. According to different specifications, our instrument was significant at the 1% level with F-statistics well above the rule-of-thumb threshold of 10 suggested by Staiger and Stock (1997) [56], reaching 315.78. Taking a fixed-effect approach, the average of *FESTs* was strong and positive determinants of FESTs, *Rfi*, and *Rde*. For instance, with one percentage increase in *FESTs*, FESTs will increase by 0.712% and *Rfi* and *Rde* will increase by 0.776% on average.

### 5.3. GMM Analysis

We used the system generalized method of moments (GMM) estimator to provide another robustness check because the endogeneity issue might also affect the analysis of panel data using fixed effects even after the use of external instruments for identification. There are three main reasons for adopting this method: Firstly, because we used annual data to measure the degree of environmental pollution, considering that environmental quality may have a hysteresis effect to some extent, this will affect the interpreted variable and the random disturbance term. Secondly, the cities themselves may have unobservable fixed effects. If these unobservable fixed effects are related to the explanatory variables, they will affect the consistency of the estimates. Thirdly, in the indicators that we used to measure social economy, there is a possibility of mutual determination. Therefore, it was important to control the potential joint endogeneity of these explanatory variables.

As shown in Table 7, all the tests indicated that the system GMM method is valid, and we found that FESTs still have a positively and statistically significant effect on air pollution in the short run, with all coefficients significant at the 1% level. The results indicate that current pollutants will drop significantly in the next period, suggesting that there is a strong inertia trend in FESTs. More importantly, based on the coefficient of FESTs in the dynamic model, the long-run effect of FESTs on the environment was consistent with the result of using the first order lag of Tec and average of Tec. This further proves that the government’s FESTs can reduce environmental pollutants.

### 5.4. Heterogeneity across Regions

Given China’s vast area and diverse economic development levels, pollution may vary substantially across regions. Lin (2017) has shown that compared to eastern and central China, western China tends to have more heavy industries, hence more pollution [57]. To further test our hypotheses, we divided the sample cities into three cohorts, i.e., western, central, and eastern China. Then, the regressions were re-run on each of the three subsamples. The results are reported in Table 8.

As shown in Table 8, in eastern China, FESTs were negatively related with SO_2_ pollution, significant at the 1% level; in central China, FESTs were negatively related with PM_2.5_ and WP, significant at the 5% and 10% levels, respectively; and in western China, FESTs were negatively related with SO_2_ and WP, both significant at the 1% level. The control variables show qualitatively similar results as those in the baseline regression. Thus, environmental pollution takes different forms in different regions. First, judging from the size of the coefficients, the western region demonstrates larger impacts of FESTs on pollution than the eastern region. This is because in the western region where local economies, innovations, and social welfare systems are all under-developed, FESTs represent a bigger share of the total spending on technologies and innovations. While in the eastern region where private sectors are strong enough to out-spend local governments in technologies and innovations, the law of diminishing marginal returns implies that the optimization effect of local FESTs is relatively small. Second, the benefits brought by FESTs in the western region are more likely to be technological advancements such as in patents. In contrast, the benefits in the eastern region are more likely to be economic structural optimizations.

### 5.5. Heterogeneity across Sub-Period Analysis

Although the environmental protection efforts of both China’s central and local governments have been increasing over time, these efforts had gone through several dramatic changes. Based on a thorough review of governments’ policies announcements, we have identified two major environmental protection policy changes. One happened after the 2008 financial crisis, and the other happened in 2013. The results are presented in Table 9.

We found significant differences in the estimated coefficients and significance of the main variables in different time periods. Between 2004 and 2008, the coefficient of FESTs on PM_2.5_ was negative and significant at the 10% level, while the coefficient on WP was positive and significant at the 1% level. During this period, FESTs alleviated air pollution, but worsened water pollution. Between 2009 and 2012, and after 2013, FESTs reduced SO_2_, but showed no significant impact on water pollution.

During the recent years, the metrics that China’s central government uses to evaluate local government official performances have been shifted from GDP-centric ones to more diversified ones. This shift has several implications for environmental protections. First, the economic growth incentives resulting from performance competition have declined, and the increase in pollution emissions caused by growth competition has slowed. Second, in recent years, the central government has set a red line for the environmental protection of local governments. For example, the central government issued documents in 2015 to incorporate environmental resource protection into the appointment of local government leading cadres, promoted the evaluation system, and implemented a lifelong accountability system.

The central government’s environmental pressure on local governments has inhibited local government pollution discharge competition to a certain extent. At the same time, the central government’s special fiscal investment has increased each year, alleviating local government’s environmental funding gap. Promoting innovation-driven and transformational development has become the focus of a new round of development in each province. Local governments have expanded environmental protection review of new projects, and the environmental impact of industrial structure upgrading has gradually emerged.

## 6. Conclusions and Policy Implications

This paper focuses on the performance of local governments as public service providers in pollution control and environmental governance. We present comprehensive theoretical and empirical examination of fiscal expenditure on the environmental pollution. We adopted 260 prefecture-level cities data during the 2004–2015 period, to assess how FESTs affected environmental pollution. The results showed that, for every 1% increase in FESTs, SO_2_ emissions were reduced by 5.293%, but the FESTs could not alleviate PM_2.5_ concentration or wastewater emission. This may be because external influences allow local governments to choose direct capital expenditures to promote economic growth and provide less public service for non-productive expenditure.

Moreover, we found that FESTs promoted R&D expenditure and impeded fixed asset investments, which together led to a reduction of the total pollution. The increase in FESTs could provide good external support for enterprises’ production-technology innovation and introduction of clean production technology while reducing the demand for polluting resources. We also identified the effects of FESTs on improving the environment by strengthening environmental regulation. The increase in FESTs show that the government recognizes the importance of environmental regulations, strengthens the supervision of enterprises, and increases the cost of producing polluting products, thereby effectively reducing investment in fixed assets.

To address potential endogeneity and introduce dynamic mechanisms, our study adopted the fixed effect model, the 2SLS method, and the GMM method, which can greatly reduce the omitted variable biases to ensure the accuracy of the results. By using tests that included alternative econometric specification and using the instrumental variable method to overcome possible endogeneity, we proved that the results were robust to a series of tests. To summarize, our findings confirmed the effectiveness of government pollution control tactics.

Our findings reinforce understanding of environmental impact of FESTs for policymakers of developing countries. These results may have important implications given the current emphasis on fiscal spending to palliate the effects of the COVID-19 pandemic. The central Chinese government also emphasizes FESTs, social spending, and environmental protection. However, under the inter-regional competition framework, local governments are more willing to inject limited financial resources into infrastructure, which plays a significant role in crowding out those FESTs with long development cycles and obvious externalities. Logically, local governments will invest fiscal funds directly in areas where performance and economic growth are prominent or in areas where investment returns are attractive, thereby reducing spending on public services such as science and technology, health care, and environmental protection. The institutional dilemma behind China’s fiscal expenditure structure, namely, political promotion incentives and performance evaluation standards centered on GDP growth, has led local governments to focus on economic growth.

Based on the above analysis, we propose the following suggestions:

Due to the problem of market failure and free-riders, it is necessary to strengthen the emphasis on environmental costs and ecological benefits in the assessment system and increase the proportion of “green GDP” in GDP [5]. The institutional dilemma behind China’s fiscal expenditure structure, namely, political promotion incentives and performance evaluation standards centered on GDP growth, has led local governments to focus on economic growth. FESTs should be regarded as an important positive indicator when conducting performance evaluation of local officials because FESTs constitute the most direct innovation for the country [27,58]. Empirical studies generally support the idea that FESTs provide incentives or leverage for enterprises’ own R&D investment [59,60]. In addition, FESTs can play a role in guiding funds because they help to attract more external private investment, thereby contributing to the optimization of technical structure. Finally, FESTs can eliminate the R&D risks for those innovation activities with long development cycles and uncertainties.

On the other hand, regarding the other control variables, the inverse U-shaped curve shows that pollution emissions caused by GDP growth competition have declined. As the biggest emerging market, China has been experiencing a transition towards high value orientation. However, serious environmental pollution is a threat to China’s sustainable development. Besides that, the environmental regulations, economic structure, and trade openness are conductive to reducing environmental pollution. Thus, the government should further widen the openness to foreign capital and deepen economic reforms which helpful to stimulate economic growth and reduce environment pollution. Lastly, government should motivate corporations to adopt cleaner technologies through subsidies and stricter environmental regulations. This approach could also be a valuable strategy to governments outside China.

## Figures and Tables

**Table 1:** Variable definitions and constructions.

Variable	Construction
*PM_2.5_*	Natural logarithm of PM_2.5_ concentration (μg/m^3^)
*SO_2_*	Natural logarithm of SO_2_ emission (ton)
*WP*	Natural logarithm of Wastewater emission (10^4^ ton)
*DPM_2.5_*	Ratio of PM_2.5_ concentration to GDP (μg/m^3^/10^8^ Yuan)
*DSO_2_*	Ratio of SO_2_ emission to GDP (ton/10^8^ Yuan)
*DWP*	Ratio of wastewater emission to GDP (ton/10^4^ Yuan)
*Rfi*	Ratio of fixed asset investments to total GDP
*Rde*	Ratio of R&D expenditure to total GDP
*FESTs*	Ratio of fiscal expenditure on ST to total government expenditure
*Pgdp*	Natural logarithm of real GDP to total population (Yuan)
*Pgdp* ^2^	The squared term of per capita GDP (Yuan)
*Es*	Ratio of the gross product in secondary industry to GDP
*Eq*	Green coverage rate of built-up areas
*Fdi*	Ratio of actual foreign investment to GDP
*Trade*	Ratio of the sum of export and import to GDP
*Fd*	Ratio of loan balance in financial institutions to GDP
*Ud*	Ratio of non-agricultural population to total population

**Table 2:** Summary statistics.

Variable	Obs.	Mean	Std.	Min	Max
*PM_2.5_*	2980	4.3883	0.8034	3.1245	7.4343
*SO_2_*	2961	3.7259	0.9523	0.5867	5.4455
*WP*	2966	8.5090	0.9616	5.7333	10.9154
*DPM_2.5_*	2980	−2.4094	1.0980	−5.0114	0.6282
*DSO_2_*	2961	3.8321	1.0693	0.3667	6.2307
*DWP*	2966	1.7070	0.8644	−0.3971	3.8550
*FESTs*	2979	0.6264	0.2348	0.2032	1.3003
*Rde*	2980	0.0287	0.0407	0.0006	0.3290
*Tec*	2980	0.0115	0.0110	0.0010	0.0565
*Pgdp*	2977	10.1194	0.7360	8.4828	11.7925
*Pgdp* ^2^	2977	102.9439	14.8994	71.9581	139.0624
*Es*	2980	49.6156	10.0335	22.7600	73.4400
*Eq*	2980	0.3728	0.0819	0.0959	0.6246
*Fdi*	2966	0.0212	0.0208	0.0003	0.1017
*Trade*	2980	0.2076	0.3501	0.0024	2.2379
*Fd*	2980	0.7716	0.4420	0.2638	2.7118
*Ud*	2937	0.7372	0.3358	0.1188	1.0139

**Table 3:** Fiscal expenditures on science and technology (FESTs) and environmental pollution.

Variable	PM_2.5_			SO_2_ Emission			Water Pollution		
	(1)	(2)	(3)	(4)	(5)	(6)	(7)	(8)	(9)
*FESTs*	−6.8567 ***	−4.5292 *	−5.3288 **	−7.4965 ***	−5.0758 ***	−5.3172 ***	0.4903	0.7504	0.2807
	(2.2052)	(2.4540)	(2.5155)	(1.6294)	(1.8115)	(1.8540)	(1.3694)	(1.5231)	(1.5585)
*Pgdp*		1.0932 **	1.3668 **		1.2195 ***	0.7707 *		0.0148	0.2700
		(0.5329)	(0.6151)		(0.3934)	(0.4533)		(0.3307)	(0.3811)
*Pgdp* ^2^		−0.0499 *	−0.0609 **		−0.0622 ***	−0.0453 **		0.0078	−0.0018
		(0.0271)	(0.0298)		(0.0200)	(0.0220)		(0.0168)	(0.0185)
*Es*			−0.0016			0.0083 ***			−0.0034
			(0.0040)			(0.0029)			(0.0025)
*Eq*			−0.0577			−0.1229			0.3180 **
			(0.2152)			(0.1586)			(0.1333)
*Fdi*			0.1177			−0.8440			−1.1474 *
			(1.0974)			(0.8088)			(0.6799)
*Trade*			−0.8899			−0.0912			−0.1804
			(0.5427)			(0.4000)			(0.3362)
*Cons.*	4.1009 ***	−1.7419	−3.1923	3.8513 ***	−2.0984	0.2892	8.5071 ***	7.6886 ***	6.2503 ***
	(0.0385)	(2.6797)	(3.1120)	(0.0285)	(1.9781)	(2.2936)	(0.0239)	(1.6632)	(1.9281)
*City-FE*	Yes	Yes	Yes	Yes	Yes	Yes	Yes	Yes	Yes
*Year-FE*	Yes	Yes	Yes	Yes	Yes	Yes	Yes	Yes	Yes
*Obs.*	2548	2548	2548	2548	2548	2548	2548	2548	2548
*adj. R* ^2^	0.4204	0.4212	0.4210	−0.0294	−0.0259	−0.0231	−0.1050	−0.1021	−0.0988

Robust standard errors are in parenthesis; *, **, and *** denote 10%, 5%, and 1% significance levels, respectively.

**Table 4:** Mechanism tests: Fixed asset investments versus R&D.

Variable	$\mathrm{Fixed}\;\mathrm{Asset}\;\mathrm{Investments}\;(Rfi_{t+1})$			$R\text{\&}D\;\mathrm{Expenditure}\;(Rdec_{t+1})$		
	(1)	(2)	(3)	(1)	(2)	(3)
*FESTs*	−4.4253 ***	−3.7761 ***	−2.7010 ***	3.2139 ***	2.4474 **	2.3953 *
	(0.4347)	(0.4448)	(0.4622)	(1.1369)	(1.1694)	(1.2375)
*Pgdp*		−0.0677 ***	−0.0837 ***		−0.2195 ***	−0.2219 ***
		(0.0234)	(0.0238)		(0.0614)	(0.0637)
*Es*		0.0051 ***	0.0046 ***		−0.0008	−0.0013
		(0.0008)	(0.0008)		(0.0020)	(0.0021)
*Fd*			−0.0076			−0.0243
			(0.0183)			(0.0489)
*Ud*			0.0981 ***			0.1528 **
			(0.0280)			(0.0750)
*Fdi*			1.4287 ***			−1.3777 **
			(0.2127)			(0.5695)
*Trade*			0.0360			−0.0281
			(0.0298)			(0.0799)
*Cons.*	0.4628 ***	0.8496 ***	0.9519 ***	0.4628 ***	0.8496 ***	0.9519 ***
	(0.0076)	(0.2026)	(0.2110)	(0.0076)	(0.2026)	(0.2110)
*City−fixed effect*	Yes	Yes	Yes	Yes	Yes	Yes
*Year−fixed effect*	Yes	Yes	Yes	Yes	Yes	Yes
*Obs.*	2631	2628	2578	2631	2628	2578
adj. *R*^2^	0.5205	0.5291	0.5384	−0.0659	−0.0590	−0.0590

Robust standard errors are in parenthesis; *, **, and *** denote 10%, 5%, and 1% significance levels, respectively.

**Table 5:** FESTs and environmental pollution intensity.

Variable	PM_2.5_/GDP			SO_2_ Emission/GDP			Water Pollution/GDP		
	(1)	(2)	(3)	(4)	(5)	(6)	(7)	(8)	(9)
*FESTs*	−4.5502 **	−4.8751 *	−5.8841 **	−5.6707 ***	−5.6626 ***	−6.1360 ***	0.4446	−0.8728	−1.4002
	(2.2690)	(2.5187)	(2.5793)	(1.6518)	(1.8211)	(1.8671)	(1.3969)	(1.5472)	(1.5774)
*Pgdp*		0.1150	0.8436		0.3403	0.5260		−0.4466	0.2662
		(0.5469)	(0.6306)		(0.3955)	(0.4565)		(0.3360)	(0.3857)
*Pgdp2*		−0.0264	−0.0552 *		−0.0421 **	−0.0502 **		0.0068	−0.0206
		(0.0278)	(0.0306)		(0.0201)	(0.0221)		(0.0171)	(0.0187)
*Es*			−0.0082 **			−0.0008			−0.0105 ***
			(0.0041)			(0.0030)			(0.0025)
*Eq*			0.0034			−0.0906			0.3680 ***
			(0.2206)			(0.1597)			(0.1349)
*Fdi*			0.0581			−0.9539			−0.9042
			(1.1252)			(0.8145)			(0.6881)
*Trade*			−1.0218 *			−0.1876			−0.2300
			(0.5565)			(0.4028)			(0.3403)
*Cons.*	−2.0151 ***	−0.7872	−4.6309	4.6261 ***	5.1245 **	4.2001 *	2.3933 ***	5.9659 ***	2.1247
	(0.0397)	(2.7503)	(3.1909)	(0.0289)	(1.9886)	(2.3098)	(0.0244)	(1.6895)	(1.9514)
*City−FE*	Yes	Yes	Yes	Yes	Yes	Yes	Yes	Yes	Yes
*Year−FE*	Yes	Yes	Yes	Yes	Yes	Yes	Yes	Yes	Yes
*Obs.*	2548	2548	2548	2548	2548	2548	2548	2548	2548
*adj. R2*	0.4252	0.4289	0.4298	0.5741	0.5825	0.5822	0.5811	0.5856	0.5899

Robust standard errors are in parenthesis; *, **, and *** denote 10%, 5%, and 1% significance levels, respectively.

**Table 6:** 2SLS estimation using the average of FESTs at the same province as an instrumental variable.

Variable	First Stage	Second Stage						First Stage	Second Stage	
		PM_2.5_	SO_2_	WP	PM_2.5/_GDP	SO_2_/GDP	WP/GDP		Rfi	Rde
*IV*	0.7116 ***							0.7764 ***		
	(0.0246)							(0.0254)		
*FESTs*		−17.7296 ***	−14.1867 ***	3.1529	−18.0619 ***	−13.4508 ***	−11.8020 ***		−4.5273 ***	1.0377 ***
		(4.3266)	(3.4222)	(2.8591)	(4.4276)	(3.4772)	(2.8648)		(0.8338)	(0.1955)
*Pgdp*	−0.0690 ***	0.0250	1.1076 **	0.7404 *	−0.6320	0.9016 *	0.7172 *	0.0005	−0.0598 ***	−0.0244 ***
	(0.0040)	(0.6209)	(0.4923)	(0.4113)	(0.6354)	(0.5002)	(0.4121)	(0.0009)	(0.0223)	(0.0052)
*Pgdp2*	0.0034 ***	0.0074	−0.0612 **	−0.0243	0.0084	−0.0799 ***	−0.0519 ***			
	(0.0002)	(0.0303)	(0.0240)	(0.0201)	(0.0310)	(0.0244)	(0.0201)			
*Es*	0.0000	0.0001	0.0096 ***	−0.0046 **	−0.0045	0.0024	−0.0102 ***	−0.0001 ***	0.0055 ***	0.0002
	(0.0000)	(0.0035)	(0.0028)	(0.0023)	(0.0036)	(0.0028)	(0.0023)	(0.0000)	(0.0007)	(0.0002)
*Eq*	0.0008	0.0242	−0.0796	0.5231 ***	−0.0668	−0.1412	0.4825 ***			
	(0.0015)	(0.1922)	(0.1526)	(0.1272)	(0.1967)	(0.1550)	(0.1275)			
*Fdi*	−0.0151 **	0.1663	−0.5439	−1.1979 *	1.0828	−0.2037	−0.6059	−0.0321 ***	2.2974 ***	0.2069 ***
	(0.0076)	(0.9586)	(0.7726)	(0.6431)	(0.9810)	(0.7850)	(0.6443)	(0.0078)	(0.2055)	(0.0482)
*Trade*	−0.0051 ***	−0.1379	0.1655 *	0.0763	0.1458	0.5244 ***	0.2603 ***	−0.0052 ***	0.0280	0.1049 ***
	(0.0009)	(0.1199)	(0.0951)	(0.0794)	(0.1227)	(0.0967)	(0.0795)	(0.0010)	(0.0261)	(0.0061)
*Fd*								0.0005	0.0152	0.0285 ***
								(0.0007)	(0.0173)	(0.0041)
*Ud*								−0.0107 ***	0.0914 ***	−0.0052
								(0.0010)	(0.0281)	(0.0066)
*Cons.*	0.3475 ***	3.2289	−1.8123	3.6993 *	3.4752	2.9315	0.5826	0.0072	0.6136 ***	0.1884 ***
	(0.0203)	(3.1435)	(2.4918)	(2.0822)	(3.2169)	(2.5319)	(2.0864)	(0.0077)	(0.1968)	(0.0461)
*City-fixed effect*	Yes	Yes	Yes	Yes	Yes	Yes	Yes	Yes	Yes	Yes
*Year-fixed effect*	Yes	Yes	Yes	Yes	Yes	Yes	Yes	Yes	Yes	Yes
*Obs.*	2937	2937	2918	2923	2937	2918	2923	2894	2893	2894
*F-statistic*	315.78							279.53		
*adj. R2*	0.6811	0.4801	0.0868	0.0279	0.4947	0.6248	0.6565	0.6578	0.6196	0.1732

Robust standard errors are in parenthesis; *, ** and *** denote 10%, 5% and 1% significance levels, respectively.

**Table 7:** Robust check: Dynamic models with dynamic method of moments (GMM) estimator.

Variable	PM_2.5_	SO_2_	WP	PM_2.5_/GDP	SO_2_/GDP	WP/GDP	Rfi	Rde
*L.y*	0.5613 ***	0.5787 ***	0.9132 ***	0.7427 ***	0.5410 ***	0.7951 ***	0.8288 ***	0.0907 ***
	(0.1944)	(0.0801)	(0.0724)	(0.1443)	(0.0752)	(0.0730)	(0.0522)	(0.0073)
*FSETs*	−4.2196	−4.4336 **	−2.2346	−7.7796 **	−9.0827 ***	−4.7616 **	−2.3766 ***	0. 6900 ***
	(3.3596)	(2.1870)	(1.5231)	(3.6061)	(2.3380)	(2.2668)	(0.7457)	(0.1984)
*Pgdp*	−0.3251	−0.2020	−0.0178	−0.8146	−0.4049	−0.0853	−0.0493 ***	−0.0111 ***
	(0.6200)	(0.3929)	(0.2571)	(0.6859)	(0.3840)	(0.2475)	(0.0072)	(0.0018)
*Pgdp* ^2^	0.0164	0.0218	0.0009	0.0300	0.0130	−0.0026		
	(0.0306)	(0.0189)	(0.0125)	(0.0354)	(0.0190)	(0.0128)		
*Es*	0.0010	0.0076 ***	0.0017 *	0.0015	0.0116 ***	0.0033 ***	0.0011 ***	0.0003 ***
	(0.0020)	(0.0018)	(0.0009)	(0.0026)	(0.0023)	(0.0013)	(0.0003)	(0.0001)
*Eq*	−0.0439	−0.0893	−0.0610	−0.1113	−0.1241	0.1272		
	(0.2620)	(0.1553)	(0.1282)	(0.2796)	(0.1511)	(0.1154)		
*Fdi*	0.4828	−0.9553	0.4933	0.7435	−1.3884 **	0.8069 **	0.8053 ***	0.1932 ***
	(0.9834)	(0.6493)	(0.4444)	(1.0958)	(0.6829)	(0.3934)	(0.1546)	(0.0286)
*Trade*	−0.0458	−0.0690 *	0.0265	0.0091	−0.0602	0.0633 ***	−0.0367 ***	0.0217 ***
	(0.0664)	(0.0388)	(0.0322)	(0.0786)	(0.0410)	(0.0245)	(0.0109)	(0.0020)
*Fd*							0.0154 ***	0.0079 ***
							(0.0052)	(0.0015)
*Ud*							0.0465 **	−0.0111 **
							(0.0186)	(0.0054)
*City−fixed effect*	Yes	Yes	Yes	Yes	Yes	Yes	Yes	Yes
*Year−fixed effect*	Yes	Yes	Yes	Yes	Yes	Yes	Yes	Yes
*Obs.*	2621	2587	2596	2621	2587	2596	2582	2582
*AR(2)*	0.262	0.378	0.238	0.406	0.384	0.736	0.787	0.647
*Sargan*	0.690	0.529	0.687	0.753	0.276	0.380	0.215	0.322

The instrumental variables are the 1–3 lags of Tec; L.y represent the first order lag of dependent variables; Robust standard errors are in parenthesis; *p*-values of AR(2) and Sargan tests are provided; *, ** and *** denote 10%, 5% and 1% significance levels.

**Table 8:** Robust check: FESTs and environmental pollution at different regions.

Variable	PM_2.5_			SO_2_ Emission			Water Pollution		
	Eastern	Central	Western	Eastern	Central	Western	Eastern	Central	Western
*FESTs*	2.4409	−7.7885 **	1.8038	−6.5693 ***	3.0764	−31.1391***	2.5518	−4.3892 *	−23.5495 ***
	(2.4621)	(3.7910)	(8.5015)	(2.3661)	(2.6941)	(7.3036)	(1.7065)	(2.4183)	(6.4777)
*Pgdp*	0.1683	1.9890 **	0.4715	1.1970	1.7089 ***	−0.3618	0.0996	1.0639 *	0.1263
	(0.7827)	(0.9264)	(1.1527)	(0.7556)	(0.6583)	(0.9881)	(0.5452)	(0.5909)	(0.8764)
*Pgdp2*	−0.0100	−0.0779 *	−0.0229	−0.0701 *	0.1023 ***	0.0367	−0.0079	−0.0402	0.0340
	(0.0382)	(0.0435)	(0.0572)	(0.0368)	(0.0309)	(0.0490)	(0.0266)	(0.0278)	(0.0435)
*Es*	0.0061	−0.0107 *	0.0018	0.0335***	0.0086 *	−0.0023	0.0019	0.0013	−0.0235 ***
	(0.0053)	(0.0064)	(0.0068)	(0.0051)	(0.0045)	(0.0058)	(0.0036)	(0.0041)	(0.0051)
*Eq*	0.0310	−0.4688	0.1096	0.0576	−0.0753	−0.0060	0.1499	1.0638 ***	0.1073
	(0.2892)	(0.3294)	(0.3774)	(0.2775)	(0.2341)	(0.3237)	(0.2003)	(0.2101)	(0.2871)
*Fdi*	2.8367 ***	−3.1855	1.7264	−0.8157	−1.0385	3.7389	−1.0517	−0.6959	−2.0280
	(0.9928)	(2.0591)	(3.6557)	(0.9764)	(1.4634)	(3.1531)	(0.7040)	(1.3135)	(2.7966)
*Trade*	−0.0841	0.0299	0.0329	0.0618	0.5060 ***	−0.1198	0.0102	0.2752 *	0.3401
	(0.1226)	(0.2478)	(0.4284)	(0.1176)	(0.1761)	(0.3681)	(0.0850)	(0.1581)	(0.3265)
*Cons.*	2.9358	−6.8341	1.4701	−2.9103	−3.9664	4.0137	8.4677 ***	1.5069	5.1229
	(3.9771)	(4.8454)	(5.6887)	(3.8372)	(3.4435)	(4.8766)	(2.7691)	(3.0909)	(4.3252)
*City−FE*	Yes	Yes	Yes	Yes	Yes	Yes	Yes	Yes	Yes
*Year−FE*	Yes	Yes	Yes	Yes	Yes	Yes	Yes	Yes	Yes
*Obs.*	1142	1173	648	1128	1173	643	1133	1173	643
*adj. R2*	0.4924	0.4530	0.6195	0.1784	0.1066	0.0687	0.0433	0.0745	0.0984

Cities are classified into different regions according to the 2007 China Statistical Yearbook; Robust standard errors are in parenthesis; *, ** and *** denote 10%, 5% and 1% significance levels, respectively.

**Table 9:** Robust check: Subperiod analysis.

Variable	PM_2.5_			SO_2_ Emission			Water Pollution		
	2004–2008	2009–2012	2013–2015	2004–2008	2009–2012	2013–2015	2004–2008	2009–2012	2013–2015
*FESTs*	−0.9012 *	−14.1514	−3.8133	−1.5555	1.5647	−12.4666 **	5.2166 ***	−0.8577	−2.6431
	(0.4666)	(9.5873)	(8.5668)	(2.7030)	(4.5226)	(6.3079)	(2.0144)	(4.6211)	(2.6120)
*Pgdp*	0.3342 **	3.0998	−3.7334	1.7099 *	1.2961	−13.6882***	2.7029 ***	1.3745	0.4302
	(0.1666)	(2.7105)	(5.0034)	(0.9595)	(1.3006)	(3.6841)	(0.7192)	(1.3308)	(1.5248)
*Pgdp* ^2^	−0.0142 *	−0.1585	0.1489	0.0966 **	−0.0731	0.6403***	0.1318 ***	−0.0438	−0.0214
	(0.0085)	(0.1320)	(0.2361)	(0.0492)	(0.0633)	(0.1739)	(0.0368)	(0.0647)	(0.0720)
*Es*	0.0016 **	0.0507 ***	0.0032	0.0141 ***	0.0036	−0.0032	−0.0043	0.0180 **	0.0077
	(0.0008)	(0.0175)	(0.0172)	(0.0046)	(0.0083)	(0.0127)	(0.0035)	(0.0084)	(0.0053)
*Eq*	−0.0596	0.4894	0.7477	−0.1618	−0.0477	−0.4524	0.2506	0.3452	0.0922
	(0.0387)	(0.5793)	(0.8888)	(0.2231)	(0.2725)	(0.6544)	(0.1672)	(0.2778)	(0.2708)
*Fdi*	0.4559 **	1.0655	1.9811	0.7685	2.0311	−2.9140	−0.7658	−2.8534	−0.1859
	(0.2076)	(4.1206)	(3.8174)	(1.1990)	(2.0490)	(2.8109)	(0.8961)	(2.0921)	(1.1632)
*Trade*	0.0015	1.0952 *	0.2219	0.2132	0.3107	−0.0473	0.3623 **	−0.0703	0.0131
	(0.0345)	(0.6495)	(0.3657)	(0.1992)	(0.3130)	(0.2692)	(0.1488)	(0.3142)	(0.1114)
*Cons.*	2.2564 ***	−13.6826	27.7690	−4.5676	−2.1419	77.0043***	−5.0546	−0.0436	5.9947
	(0.8016)	(13.9124)	(26.3706)	(4.6178)	(6.6760)	(19.4174)	(3.4608)	(6.8330)	(8.0364)
*City−FE*	Yes	Yes	Yes	Yes	Yes	Yes	Yes	Yes	Yes
*Year−FE*	Yes	Yes	Yes	Yes	Yes	Yes	Yes	Yes	Yes
*Obs.*	1195	1021	747	1193	1004	747	1195	1008	746
adj. *R*^2^	0.4835	0.3841	0.6552	0.0932	0.0151	0.0951	0.0722	0.0223	0.0527

Robust standard errors are in parenthesis; *, ** and *** denote 10%, 5% and 1% significance levels, respectively.

## Appendix A. Theory Models

We constructed a conceptual framework to investigate the relationship between FEST and the environmental pollution based on the neoclassical Solow–Swan model and the aggregate economy-environment interaction model. Following Hua et al. (2018), Keeler et al. (1971), López et al. (2011), and Hritonenko and Yatsenko (1999) [3,4,60,61], we constructed the following public-good fiscal spending model:

(A1) $$U\left(C,P\right)=lnC-\gamma \frac{P_{t}^{1+\lambda }}{1+\lambda }$$

where $U\left(C,P\right)$ is a social utility function combined the utility of consumption C and the negative utility of air pollutant emission P; *lnC* and $\frac{P_{t}^{1+\lambda }}{1+\lambda }$ (with γ > 0) are logarithmic utility function and power utility function, respectively, and both satisfying Inada conditions. γ > 0 indicates society’s magnanimity of environmental pollutant. Thus, we obtain the associated current-valued objective function as follow:

(A2) $$Max\int_{0}^{T}e^{-rt}\left[lnC_{t}-\gamma \frac{P_{t}^{1+\lambda }}{1+\lambda }\right]dt$$

subject to the following constrains:

(A3) $$Q_{t}=sK^{\alpha }$$

(A5) $$P_{t}=\eta \left(Q_{t}-\frac{F}{\mu }\right)+\epsilon$$

(A6) $$Q_{t}=I_{t}+C_{t}+F_{t}$$

(A7) $${\dot{K}}_{t}=I_{t}-\delta K_{t}$$

(A8) $$K_{0}=k_{0},\;K_{T}=k_{T};F_{0}=f_{0},\;F_{T}=f_{T}$$

where $P_{t}$ is the function of total environmental pollution. $Q_{t}$ is the final product including investment *I*, consumption *C* and public-good fiscal expenditure *F*. *C* and *I* are independent control variables and *F* is a state variable depending on *C* and *I*. ${\dot{K}}_{t}$ is the motion equation that picture the movement of capital *K* as a function of *I* and depreciates at a constant rate δ > 0. Constraint (4) contains boundary conditions. The current-valued Hamiltonian of this study is:

(A9) $$H\left(C,K,F,t\right)=e^{-rt}\left\{lnC-\gamma \frac{{\left[\eta \left(SK^{\alpha }-\frac{F}{\mu }\right)+\varepsilon \right]}^{1+\lambda }}{1+\lambda }\right\}+\omega \left(SK^{\alpha }-C-F-\delta K\right)$$

where *H* is strictly concave and differentiable. ω is associated with constraint (A6) and (A7). According to the Pontryagin maximum principle, the optimality conditions are as following:

(A10) $$\frac{\partial H}{\partial C}=e^{-rt}\frac{1}{C}-\omega =0$$

(A11) $$\frac{\partial H}{\partial K}=-e^{-rt}\gamma {\left[\eta \left(SK^{\alpha }-\frac{F}{\mu }\right)+\varepsilon \right]}^{\lambda }\eta S\alpha K^{\alpha -1}+\omega S\alpha K^{\alpha -1}-\omega \delta =0$$

(A12) $$\frac{\partial H}{\partial F}=e^{-rt}\gamma {\left[\eta \left(SK^{\alpha }-\frac{F}{\mu }\right)+\varepsilon \right]}^{\lambda }\frac{1}{\mu }-\omega =0$$

where Equation (A9) is the first-order extremum conditions. Equations (A10) and (A11) are Euler conditions. We adopt comparative static analysis to yield the relationship between fiscal spending and environment pollution. Then, we construct a steady-state study as follows:

(A13) $$\frac{1}{SK^{\alpha }-F-\delta K}=\frac{\gamma }{\mu }{\left[\eta \left(SK^{\alpha }-\frac{F}{\mu }\right)+\varepsilon \right]}^{\lambda }$$

where *K* is a constant. From the above steady-state equation, we propose that the impact of the local government’s FEST on the environmental pollution is negative.
